# A Novel Joint Problem of Routing, Scheduling, and Variable-Width Channel Allocation in WMNs

**DOI:** 10.1155/2014/754749

**Published:** 2014-04-02

**Authors:** Chun-Cheng Lin, Wan-Yu Liu, Chun-Hung Chou, Der-Jiunn Deng

**Affiliations:** ^1^Department of Industrial Engineering and Management, National Chiao Tung University, Hsinchu 300, Taiwan; ^2^Department of Tourism Information, Aletheia University, New Taipei City 251, Taiwan; ^3^Department of Computer Science and Information Engineering, National Changhua University of Education, Changhua 500, Taiwan

## Abstract

This paper investigates a novel joint problem of routing, scheduling, and channel allocation for single-radio multichannel wireless mesh networks in which multiple channel widths can be adjusted dynamically through a new software technology so that more concurrent transmissions and suppressed overlapping channel interference can be achieved. Although the previous works have studied this joint problem, their linear programming models for the problem were not incorporated with some delicate constraints. As a result, this paper first constructs a linear programming model with more practical concerns and then proposes a simulated annealing approach with a novel encoding mechanism, in which the configurations of multiple time slots are devised to characterize the dynamic transmission process. Experimental results show that our approach can find the same or similar solutions as the optimal solutions for smaller-scale problems and can efficiently find good-quality solutions for a variety of larger-scale problems.

## 1. Introduction

Wireless mesh networks (WMNs) are able to achieve a higher network coverage rate and a larger transmission range through massive deployment of nodes and data exchange between nodes. Aside from the conventional functions of wireless networks, mesh routers in WMNs can dynamically access, set up, and debug one another; that is, even if one of the mesh routers shuts down, the network itself can perform self-recovery and discovery. In general, the practical problems for routing, scheduling, and channel allocation in WMNs are computationally intractable, and, hence, a lot of heuristic and metaheuristic algorithms for efficiently solving the related problems have been proposed, for example, the ant simulated annealing algorithm for the routing problem [1], the approximate dynamic programming approach for link scheduling [2], the local search based algorithm for energy efficient spatial scheduling [3], and the genetic algorithm for the channel assignment problem with partially overlapping channels [4], among others.

In addition to the individual problems for routing, scheduling, and channel allocation in WMNs, the metaheuristic algorithms for the joint problems for WMNs have been investigated; for example, the joint routing and scheduling problem was solved by a genetic algorithm in [5]; the joint routing and channel allocation problem was solved by a simulated annealing algorithm [6] and a genetic algorithm in [7], among others. However, most of the previous joint problems for WMNs were associated with only two of the three concerns, and hence this paper further investigates the joint problem of routing, scheduling, and channel allocation (RSC for short) to address the network scenario more realistically. Another important issue on WMNs is the influence of signal interference on network performance, which can be lessened by using multiple radios and multiple orthogonal channels; for example, see [8, 9]. A recent work in [10] has proposed a software technique with very little overhead that can dynamically adapt the widths of multiple channels at different times so as to attain more concurrent transmissions and reduce signal interference under limited resource of wireless spectrum. Note that although the previous works for the joint RSC problem in [11, 12] have proposed to apply such a variable-width channel allocation scheme to control signal interference, some delicate constraints are not concerned in their linear programming models.

In light of the above, this paper first constructs a linear programming model that better reflects the joint RSC problem in WMNs and then designs the simulated annealing (SA) algorithm with a novel encoding mechanism to solve the problem. Our approach attempts to more realistically address how WMN operates and to rapidly identify feasible paths and schedule, as well as variable-width channel allocation of data transmission in large-scale network topologies. In addition, we further take the influence of signal interference on the channel capacity into consideration to better reflect real network situations. Our experimental results are first compared with the optimal solutions for the small-scale problems that do not consider the influence of signal interference on channel capacity, and then a comprehensive experimental analysis on more complex problems is conducted. Note that the work in [11] has proposed an SA approach for a joint routing, link scheduling, and spectrum allocation, but its linear programming model and our SA approach are different from ours.

The main contributions of this paper are stated as follows:

- the SA algorithm with a novel encoding mechanism is designed and implemented to solve a novel joint RSC problem with variable-width channel allocation scheme;
- we establish a linear programming model for the novel joint RSC problem, in which in order to be more realistic than the previous works in [11, 12], we consider that the channel capacity is influenced by the allocated channel width, transmission range, and signal interference;
- we propose an innovative solution encoding design in which the configurations of multiple time slots are devised to characterize the dynamic data transmission process in WMNs.

The rest of this paper is organized as follows.  Section 2 covers the literature review and channel allocation schemes.  Section 3 gives the solution encoding design of our SA algorithm for the concerned problem and then describes the details of the algorithm.  Section 4 briefs the designs of all experimental scenarios and their results.  Section 5 concludes this paper with future work.

## 2. Preliminaries

This section first provides reviews on related literatures, then describes the variable-width channel allocation scheme, and finally introduces the formulas on signal interference.

### 2.1. Related Work

**Figure 1:**
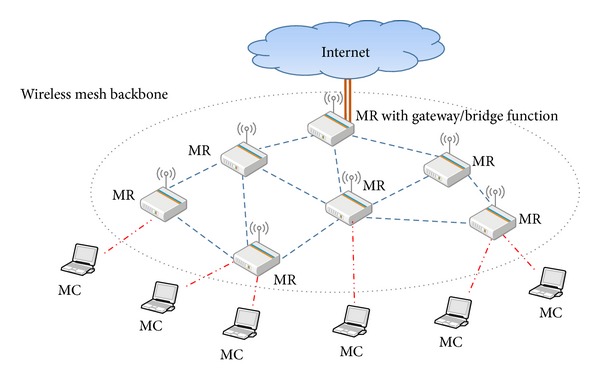
Illustration of a wireless mesh network.

The WMN is a wireless multihop technology that uses all the network devices within the transmission range as relay nodes. Based on a routing mechanism of WMN, data can be transmitted from the source node, along relay nodes on the routing path, and finally to the destination node. As shown in Figure 1, all the devices in the WMN are classified as either a mesh router (or MR) or mesh client (or MC). Besides its basic routing function, the MR in the network also serves as a gateway and bridge to transmit data to the Internet. Sessions are transmitted between MCs and MRs by utilizing the P2P mechanism as specified in the ad hoc mode. To an MC, the support for its hardware and software is far easier than an MR, because the MC does not function as a gateway and bridge. Thus, it only needs a wireless adaptor and the traffic load processed by its protocol is lighter than that of an MR. In terms of mobility, an MR is more stationary than an MC. To an MC, MRs are the mesh backbone of the network.

Most of the previous works related to this paper are dedicated to improving overall network throughput and developing new methods to address the issues on routing, scheduling, and channel allocation. The routing problem is concerned with identifying the optimal path for data transmission; for example, Li et al. [13] proposed an adaptive routing algorithm for multiple subscribers in WMNs; Draves et al. [8] applied the cumulative weighted expected transmission duration method to evaluate whether the multichannel allocation approach can effectively improve network throughput. The scheduling problem is mainly about the priority and sequence of data transmission; for example, Tang et al. [14] constructed a model that can schedule appropriately, maximize output efficiency, and control power and applied an algorithm based upon the time series theory to solve all related issues. The channel allocation problem is concerned with signal interference and concurrent transmission, and an appropriate allocation of limited spectrum resources can maximize the transmission efficiency; for example, Ko et al. [15] developed a modularized distributed channel allocation approach and made channel selection decisions based on the information acquired from the data forwarding mechanism.

As more and more studies are committed for those three problems individually, some works started discussing how the joint problems consisting of two or three individual problems can be improved at once. For instance, for joint routing and scheduling problem, Badia et al. [5] established an integer linear programming model and proposed a genetic algorithm approach for the joint routing and scheduling problem. Li et al. [16] proposed an optimization architecture for joint multipath routing and scheduling in WMNs, while Luo et al. [17] incorporated the greedy algorithm and column generation algorithm to effectively raise the overall network throughput. For the joint routing and channel allocation problem, Raniwala et al. [18, 19] used local traffic information, incorporated with distributed and centralized algorithms, to handle dynamic channel allocation and routing searches. Cheng and Yang [6] proposed a simulated annealing algorithm based upon the encoding of the path oriented mechanism and used the tree data structure to express each candidate solution for multiple paths. Valarmathi and Malmurugan [20] designed a traffic-aware and forecasting mechanism to control routing congestion and used the distributed channel allocation to avoid signal interference within the same channel.

With more complicated concerns, some works focused on the joint problem of routing, scheduling, and channel allocation problem (RSC for short). For instance, Tang and Brandt-Pearce [21] used the free space optics communication, a wireless spectrum technology, to offset insufficient spectrum resources and used a mixed-integer programming approach to solve the joint RSC problem of the two wireless technologies. Uddin et al. [11, 12] adopted the variable-width channel allocation scheme to construct a joint RSC model and solved it with the column generation algorithm and some heuristics.

### 2.2. Variable-Width Channel Allocation

**Figure 2:**
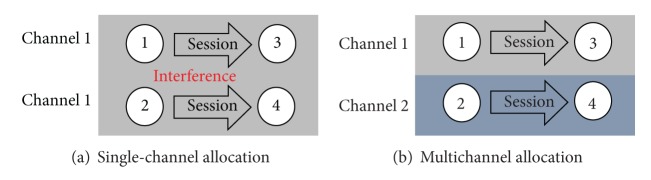
Difference between single-channel and multichannel allocations.

Channel allocation is an important topic in the study of IEEE 802.11 MAC protocols. A good channel allocation approach can drastically reduce interference for concurrent transmission, increase throughput, and reduce transmission delay [15]. The use of multiple channels may increase the complexity of channel allocation but can further lessen the influence of signal interference on network performance. The difference between single-channel and multichannel allocations [22] is illustrated in Figure 2 in which nodes 1 and 2 transmit their sessions to nodes 3 and 4, respectively. Under single-channel allocation, both of the two links are allocated with channel 1, so that nodes 1 and 2 could interfere with each other during the transmission process. In this case, it is required to set transmission priority for the two sessions. On the other hand, under multichannel allocation, the link between nodes 2 and 4 can be allocated to another channel so that the sessions with different destination nodes can be transmitted concurrently, so the channel capacity is only affected by the signal interference within a channel.

Through settings of software, MRs, as well as MCs, the variable-width channel allocation scheme can be adopted [10]. Besides the standard 20 MHz channel width allocation, different channel widths (including 5 MHz, 10 MHz, 20 MHz, and 40 MHz) for multiple channels can be set according to the transmission range as well as the number of sessions to be transmitted. Thus, the work in [10] quantified the influences of channel width on the transmission range and the number of transmitted sessions, as well as power consumption, and discovered that a smaller channel width is more appropriate for a link with less traffic load, because if a small channel width is allocated, the remaining spectrum band width can be utilized to create more orthogonal channels for other concurrent transmissions.

The work in [23] already proposed a model for the variable-width channel allocation problem and indicated that the variable-width channel allocation scheme can make a fairer and more effective use of limited spectrum resource and clearly perform superior to the conventional fixed-width channel allocation schemes. The work in [24] proposed an algorithm to suppress interference in concurrent transmission and used the variable-width channel allocation scheme to increase the number of orthogonal channels. According to the work in [24], the variable-width channel allocation scheme can improve the overall network throughput.

### 2.3. Signal Interference

Consider the transmission between two neighboring nodes *i* and *j* in which the Euclidean distance between them is *d*_*i*,*j*_. Assuming absence of interference and a simple 2-ray propagation model, the signal-to-noise ratio (SNR) at the intended receiver and the derived transmission range *D*_*w*_ are given as follows, respectively:

(1) $$\begin{array}{c} {\text{SNR}}_{i,j}=\frac{P_{o}d_{i,j}^{-\alpha }}{N_{0}W},\\ D_{w}=\sqrt[{\alpha }]{\frac{P_{o}}{N_{0}W\beta }}, \end{array}$$

where *P*_*o*_ is the transmission power; *α* is the path loss exponent; *N*_0_ is the power spectral density of thermal noise; *W* is the allocated channel width; and *β* is the required threshold that the SNR must be no less than to achieve a particular data rate. From the above equations, a larger transmission range *D*_*w*_ can be accomplished with a smaller channel width *W*. That is, if the traffic load on a link is heavier, a larger channel width should be allocated for that link.

Considering the presence of interference from other transmissions, the signal-to-interference-plus-noise ratio (SINR) at receiver *j* and the Shannon capacity of link (*i*, *j*) are, respectively, given as follows:

(2) $$\begin{array}{c} {\text{SINR}}_{i,j}=\frac{P_{o}d_{i,j}^{-\alpha }}{N_{0}W+\sum_{\alpha \;\neq \;i}P_{o}d_{a,j}^{-\alpha }},\\ C_{i,j}=W\cdot \log_{2}\left(1+\beta \right),\;\;\text{if}\;{\text{SINR}}_{i,j}\geq \beta , \end{array}$$

where *β* is the required threshold that the SINR must be no less than to achieve a particular data rate. From the above equations, a larger channel capacity can be achieved if a larger channel width *W* is allocated.

## 3. Our SA Approach to the RSC Problem

This section first describes our concerned RSC problem for WMNs, establishes a linear programming model for the problem, and covers the design of the SA algorithm, including the solution encoding mechanism, generation of the initial solution, and search of the neighboring solution.

### 3.1. Problem Description and Modelling

Consider to transmit *M* concurrent sessions in an *n*-node multihop WMN with *K* orthogonal channels, where each session *m* is transmitted from node *s*_*m*_ to node *d*_*m*_ and its traffic load is *R*_*m*_ bits for 1 ≤ *m* ≤ *M*; each channel is allocated with a spectrum band of 5, 10, 20, or 40 MHz at different times; that is, it is a variable-width channel allocation scheme. The transmission is involved with the joint problem of routing, scheduling, and channel allocation as each session may be separated into subsessions that go along different paths to its destination, and those routing paths are chosen according to the underlying schedule of different concurrent transmissions. The objective of our problem is to minimize the total system activation time for transmitting the *M* concurrent sessions while the minimum SINR requirement for transmissions is satisfied.

**Figure 3:**
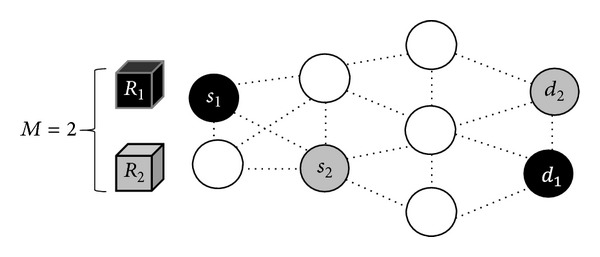
Illustration of transmitting two sessions in a 9-node network topology. The sizes of the two sessions are *R*_1_ an *R*_2_, respectively, and the pairs of their source and destination nodes are (*s*_1_, *d*_1_) and (*s*_2_, *d*_2_), respectively.

Since different channel widths may influence the transmission range, only the two neighboring nodes within the transmission range (determined by the allocated channel width) and without violating the minimum SINR requirement can communicate with each other. The link in which the two adjacent nodes can communicate with each other is called an active link. Note that the active links at different times are different. Let *E* denote the set of all possible active links. For example, in Figure 3, two sessions with sizes *R*_1_ and *R*_2_ are transmitted in a 9-node network topology; all the active links are represented by dotted lines; nodes *s*_1_ and *d*_1_ (both in black) are the source and destination nodes for session 1, while nodes *s*_2_ and *d*_2_ (both in gray) are the source and destination nodes for session 2.

**Figure 4:**
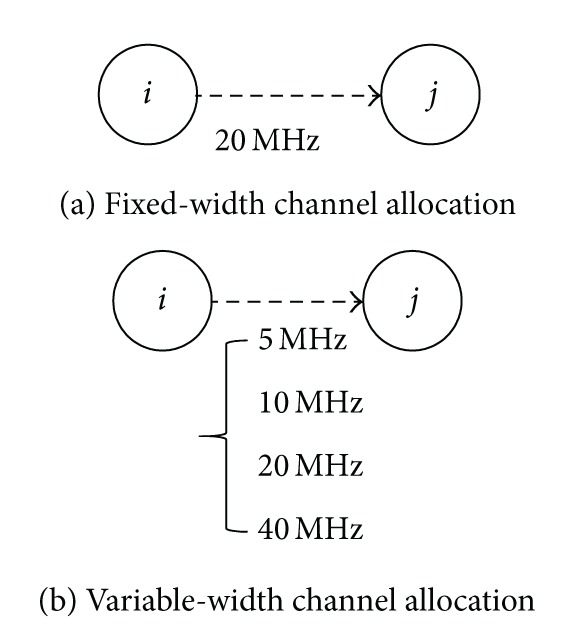
Illustration of the difference between fixed-width and variable-width channel allocation schemes.

For the variable-width channel allocation scheme, this paper continues using the setting of the total spectrum width in [11, 12]: *B* = 80 MHz. Different from the conventional fixed-width channel allocation scheme that allocates 20 MHz to all links for transmission, this paper adopts four possible channel widths: 5, 10, 20, and 40 MHz, as illustrated in Figure 4. If the allocated channel width for a link is smaller, then the transmission range is larger, and there are more orthogonal channels available to be used, but the channel capacity is smaller. On the contrary, if the allocated channel width for a link is larger, then the transmission range is smaller, and there are less remaining spectrum resources that are available to be allocated, but the channel capacity is larger.

In what follows, the linear programming model for our concerned problem is described in detail. We assume a time-division multiple access WMN, in which time is divided into *T* times slots. Note that each link is active and needs to be allocated with a channel width only when the transmission range constraint is not violated and the SINR requirement is satisfied. Hence, the multihop transmissions via active links at each time slot may take different time; that is, the durations of any two time slots may not be equal. For 1 ≤ *t* ≤ *T*, let *λ*_*t*_ denote the duration of the *t*th time slot, which is called the *t*th system activation time. The objective of our concerned problem is to minimize the total system activation time as follows:

(3) $$\begin{array}{c} \text{Minimize}\;\;\overset{T}{\underset{t=1}{\sum }}\lambda_{t}. \end{array}$$

Assume that there are *K* orthogonal channels that can be allocated within a time slot. By using the variable-width channel allocation scheme, the total spectrum width (80 MHz) can be sliced into at least 2 channels of width 40 MHz and up to 16 channels of width 5 MHz, and, hence, 2 ≤ *K* ≤ 16. Let *k* denote a particular channel. The link variable *x*_*i*,*j*_^*k*,*t*^ is defined as follows:

(4) xi,jk,t={1,if  link  (i,j)  on⁡  channel  k  is  active  at  time  slot  t;0,otherwise.

Suppose that each node is allocated with a single-radio channel; that is, each link within a time slot does not allow any traffic flow from both directions of this link simultaneously and is allocated with only one channel. Hence, the transmission constraint at each time slot is characterized as follows:

(5) ∑k=1K(∑i∈V:(i,v)∈Exi,vk,t+∑j∈V:(v,j)∈Exv,jk,t)≤1,      ∀v∈V, ∀t=1,…,T,

where *V* denotes the set of nodes in the network topology.

The spectrum band width *b* ^*k*,*t*^ allocated for channel *k* at time slot *t* is given as follows:

(6) $$\begin{array}{c} b^{k,t}=\{\begin{array}{cc} 0, & \text{if}\;\text{channel}\;k\;\text{is}\;\text{allocated}\;\text{with}\;0\;\text{MHz};\\ 5, & \text{if}\;\text{channel}\;k\;\text{is}\;\text{allocated}\;\text{with}\;5\;\text{MHz};\\ 10, & \text{if}\;\text{channel}\;k\;\text{is}\;\text{allocated}\;\text{with}\;10\;\text{MHz};\\ 20, & \text{if}\;\text{channel}\;k\;\text{is}\;\text{allocated}\;\text{with}\;20\;\text{MHz};\\ 40, & \text{if}\;\text{channel}\;k\;\text{is}\;\text{allocated}\;\text{with}\;40\;\text{MHz}. \end{array} \end{array}$$

Since the total sum of all the *K* orthogonal channels at time slot *t* cannot exceed the total spectrum width *B*, the constraint for *b* ^*k*,*t*^ is characterized as follows:

(7) $$\begin{array}{c} \overset{K}{\underset{k=1}{\sum }}b^{k,t}\leq B,\;\forall t=1,\ldots ,T. \end{array}$$

If *b* ^*k*,*t*^ = 0, then there is no spectrum band width allocated for channel *k* at time slot *t*; that is, *x*_*i*,*j*_^*k*,*t*^ must be equal to 0. Hence, we have the following constraint:

(8) $$\begin{array}{c} x_{i,j}^{k,t}\leq b^{k,t},\;\forall \left(i,j\right)\in E,\;\;\forall k=1,2,\ldots ,K. \end{array}$$

For link variable *x*_*i*,*j*_^*k*,*t*^ and channel width variable *b* ^*k*,*t*^, the allocated channel width and the active status for link (*i*, *j*) at time slot *t* are represented by *G*_*i*,*j*_^*t*^ as follows:

(9) $$\begin{array}{c} G_{i,j}^{t}=\{\begin{array}{cc} b^{k,t}, & \text{if}\;\text{link}\;\left(i,j\right)\;\text{is}\;\text{active}\;\left(\text{i}.\text{e}.,x_{i,j}^{k,t}=1\right);\\ 0, & \text{if}\;\text{link}\;\left(i,j\right)\;\text{is}\;\text{inactive}\;\left(\text{i}.\text{e}.,\overset{K}{\underset{k=1}{\sum }}x_{i,j}^{k,t}=0\right). \end{array} \end{array}$$

To linearize the definition of *G*_*i*,*j*_^*t*^ for alleviation of computing the mathematical model, three large positive constants, *M*_1_, *M*_2_, and *M*_3_, are used to construct this model:

(10) Gi,jt≤bk,t+M1(1−xi,jk,t), ∀(i,j)∈E,         ∀k=1,2,…,K,

(11) $$\begin{array}{c} G_{i,j}^{t}\leq M_{2}\overset{K}{\underset{k=1}{\sum }}x_{i,j}^{k,t},\;\forall \left(i,j\right)\in E, \end{array}$$

(12) Gi,jt≥bk,t−M3(1−xi,jk,t), ∀(i,j)∈E,         ∀k=1,2,…,K.

From the SNR equation in (1), since the allocated channel width *W* determines the maximal transmission range *D*_*W*_, we require the constraint: if the distance between two nodes exceeds this range, sessions cannot be successfully transmitted. From the above derivation, link (*i*, *j*) is allocated with channel width *G*_*i*,*j*_^*t*^, so the transmission range is *D*_*G*_*i*,*j*_^*t*^_ by (1), and, hence, we have the following constraint:

(13) di,j≤DGi,jt,   if  link  (i,j)  is  active  at  t  (i.e.,xi,jk,t=1),        ∀(i,j)∈E, ∀k=1,2,…,K.

To linearize the above inequality, we have the following:

(14) $$\begin{array}{c} d_{i,j}\leq D_{G_{i,j}^{t}}+M_{4}\left(1-x_{i,j}^{k,t}\right),\;\forall \left(i,j\right)\in E,\;\forall k=1,2,\ldots ,K, \end{array}$$

where *M*_4_ is a large positive constant.

For each active link (*i*, *j*) at each time slot *t*, if the SINR (i.e., SINR_*i*,*j*_^*t*^) is greater than the threshold *β*, the channel capacity is not affected; otherwise, the channel capacity drops. The linearized SINR equation is

(15) SINRi,jt≤Podi,j−αxi,jk,t+M5(1−xi,jk,t)N0Gi,jt+∑(a,b)∈E;a ≠ iPoda,j−αxa,bk,t, ∀(i,j)∈E,              ∀k=1,2,…,K,

where *M*_5_ is a large positive constant. Let *C*_*i*,*j*_^*t*^ denote the Shannon capacity at time slot *t*, which can be determined by the following constraint:

(16) $$\begin{array}{c} C_{i,j}^{t}\leq G_{i,j}^{t}\cdot \log_{2}\left(1+\min\left({\text{SINR}}_{i,j}^{t},\beta \right)\right). \end{array}$$

Note that the above inequality can be linearized easily.

Assuming that *f*_*i*,*j*_^*m*^ is the amount of session *m* flowing through link (*i*, *j*), the flow constraints are characterized as follows:

(17) ∑j∈V:(i,j)∈Efi,jm−∑j∈V:(j,i)∈Efj,im=0, ∀i∈V−{sm,dm},              ∀m=1,…,M,

(18) ∑j∈V:(sm,j)∈Efsm,jm−∑j∈V:(j,sm)∈Efj,smm=Rm,         ∀m=1,…,M,

(19) ∑j∈V:(dm,j)∈Efdm,jm−∑j∈V:(j,dm)∈Efj,dmm=−Rm,          ∀m=1,…,M.

Equation (17) enforces that if the concerned node *i* is neither the source node nor the destination node, each session *m* flowing into node *i* must come out eventually. Equations (18) and (19) enforce that both the total outflows of the source node and the total inflows of the destination node for session *m* must be *R*_*m*_.

Let *f*_*i*,*j*_^*m*,*t*^ be the amount of session *m* flowing through link (*i*, *j*) during time slot *t*. That is,

(20) $$\begin{array}{c} \overset{T}{\underset{t=1}{\sum }}f_{i,j}^{m,t}=f_{i,j}^{m},\;\forall \left(i,j\right)\in E,\;\;\forall m=1,\ldots ,M. \end{array}$$

Hence, the *t*th system activation time can be calculated as follows:

(21) $$\begin{array}{c} \lambda_{t}=\underset{(i,j)\in E}{\max}\left(\frac{\sum_{m=1}^{M}f_{i,j}^{m,t}}{C_{i,j}^{t}}\right),\;\forall t=1,\ldots ,T. \end{array}$$

The following constraint enforces that the total traffic load flowing through link (*i*, *j*) must not exceed the maximum capacity that this link can sustain:

(22) ∑t=0Tλt·Gi,jt·log⁡2(1+β)−∑m=1Mfi,jm≥0,           ∀(i,j)∈E.

The following constraints enforce that some variables are nonnegative:

(23) $$\begin{array}{c} f_{i,j}^{m}\geq 0,\;\;\lambda_{t}\geq 0. \end{array}$$

To summarize, the objective of our linear programming model is to minimize the total system activation time in (3), under the one-time-slot transmission constraint (5), the channel width constraints (7)–(12), the transmission range constraint (14), the SINR constraint (15), the Shannon capacity constraint (16), the one-session traffic flow constraints (17)–(19), the system activation time constraints (20)-(21), the one-link traffic flow constraint (22), and the nonzero variable constraints (23).

It is worth mentioning that the differences between our model and the previous model for the RSC problem proposed in [11, 12] are the introduction to (14)–(16) and (20)-(21). The detailed explanations are stated as follows:

- unlike the previous work, inequality (14) takes into consideration the influence of the allocated channel width on the transmission range. It enforces that the transmission range between two nodes due to the allocated channel width is no less than their Euclidean distance;
- the previous work did not consider SINR in its linear programming model. In this paper, (15) considers that different active links and their different transmission ranges result in different SINRs;
- previous work only considered the total channel capacity constraint for transmission. In this paper, the influence of signal interference on the channel capacity for each link is considered in (14)–(16), and, thus, the channel capacities for each link at each time slot may be different;
- based on the above, different capacities lead to different system activation time for each time slot. Hence, this paper introduces (20)-(21) for calculating the system activation time for each time slot.

### 3.2. Our SA Algorithm

The SA algorithm [23, 25] is an iterative improvement process inspired from simulating the annealing process and has frequently been used to solve a variety of combinatorial optimization problems, for example, periodic routing problem of a retail distribution system [26] and dynamic facility layout problem [27], among others. The basic idea of the SA is to simulate the annealing process of metal crystals to iteratively refine the solution quality and search for nearly optimal solutions. First, the encoding of any solution for the concerned problem into a system state of the metal is defined, and the relationship between the objective function of the concerned problem and the energy function of the state is specified. Next, the SA algorithm initializes the initial system state as well as some parameters on temperatures. In each iteration of the SA algorithm, the system temperature is decreased to a fixed level according an annealing schedule and a neighboring state of the current system state at the current system temperature is generated. If the energy level of this neighboring state is lower (better) than that of the current state, the neighboring state replaces the current state; otherwise, there is an acceptance probability that allows the neighboring state with a higher (worse) energy level to replace the current state. Once the current state stabilizes at the current temperature, we repeat the same annealing process until the current temperature drops below the lowest temperature. By the acceptance probability mechanism, the system can be released from the trap to constantly seek for a better local optimal solution and could therefore find a global optimal solution for the concerned problem.

**Figure 5:**
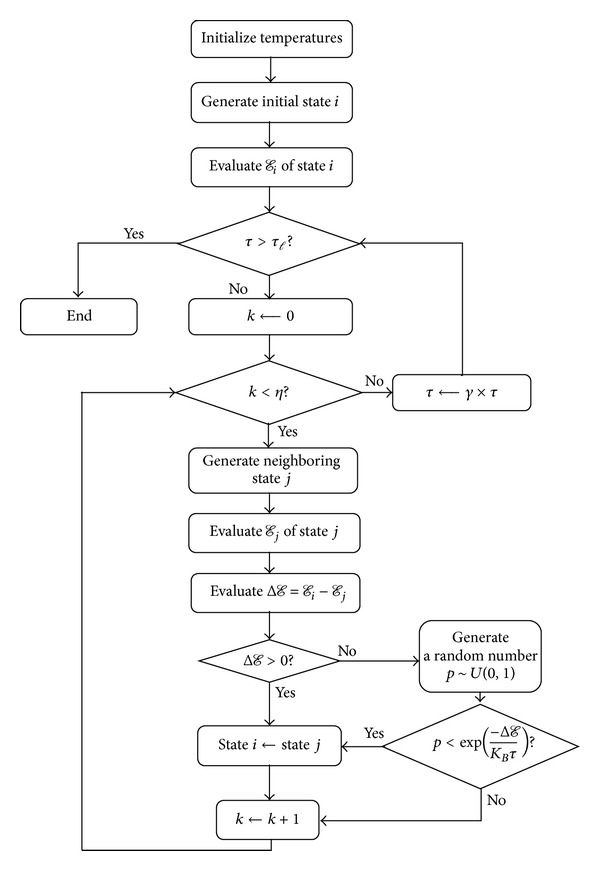
Flow chart of our SA algorithm.

The flow chart of the SA algorithm is given in Figure 5. The main steps of the SA algorithm are stated as follows:

- set the highest temperature *τ*_*h*_, the lowest temperature *τ*_*ℓ*_, and the current temperature *τ* = *τ*_*h*_;
- generate the initial state *i* and evaluate its energy level *ℰ*_*i*_;
- change the current state via perturbation to generate a new state *j*, and evaluate its energy level *E*_*j*_. State *j* is also known as the neighboring state of state *i*;
- calculate the energy difference Δ*ℰ* = *ℰ*_*i*_ − *ℰ*_*j*_ between states *i* and *j*. If Δ*ℰ* > 0, that is, the neighboring state *j* has a lower energy level, then state *j* replaces the current state *i*. Otherwise, generate a random number *p* ~ *U*(0,1) and calculate the acceptance probability *P*(Δ*ℰ*, *τ*) = exp⁡(−Δ*ℰ*/(*K*_*B*_ · *τ*)), where *K*_*B*_ is the Boltzmann constant and is usually set to 1 and *τ* is the current system temperature. If *p* < *P*(Δ*ℰ*, *τ*), then the neighboring state *j* is accepted to replace the current system state *i*; otherwise, it is rejected;
- if the maximal number *η* of iterations is not reached under the current temperature *τ*, we go back to Step (iii);
- if *τ* > *τ*_*ℓ*_, the current temperature *τ* is decreased according to the annealing schedule *τ* ← *γ* × *τ*, where *γ* ∈ (0,1), and then we go back to Step (iii); that is, the algorithm stops when *τ* ≤ *τ*_*ℓ*_.

In light of the above, we need to design the energy function for a system state and some main components of the algorithm specifically for our concerned problem. Since this paper is concerned with a minimization problem and the SA also solves for the minimal energy level, we can directly use (3) as the energy function for the system state. Hence, the remaining components that we have to design include the state encoding mechanism, the method of generating the initial state, and our neighborhood search method, which are detailed in the rest of this section.

#### 3.2.1. State Encoding

**Figure 6:**
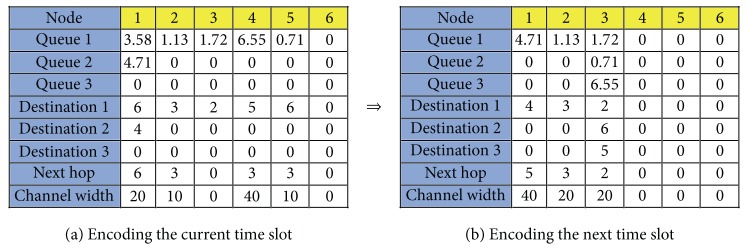
Encoding the configuration for a single time slot.

To solve our problem with the SA algorithm, we need to design a mechanism to encode the solution of the concerned problem into a system state. For our problem, the information of a system state needs the number of nodes of the network topology, the size of each session, the next hop of session transmission, and the destination node, as well as the allocated channel width. Since time is divided into multiple time slots, we first encode the configuration of a time slot as illustrated in Figure 6(a), which include the following five attributes:

- node: this records the ID of a node in the network topology;
- Queues 1–3: since we allow at most three sessions to stay at the same node at a time point, we assume that each node has a queue with three entries labeled by “Queue 1”–“Queue 3.” Each entry of the queue can store a session staying at the current node, and its value records its size. If the value is 0, it means that the queue entry is empty;
- Destinations 1–3: for *i* = 1,2, 3, the “Destination *i*” entry records the ID of the destination node of the session at “Queue *i*” of the current node;
- next hop: this records the ID of the next hop node to which the session at “Queue 1” is going to be transmitted. If the ID of the next hop node coincides with that of “Destination 1,” then the transmission of the session at “Queue 1” will be finished after this time slot;
- channel width: this records the allocated channel width for the link from the current node to the next hop; for example, a 20 MHz channel width is allocated to link (1, 6) in Figure 6(a).

To guarantee the solution feasibility, the configuration of a single time slot needs to satisfy the following constraints:

- the queue at each node is first-in-first-out, and, hence, for *i* = 1,2, if the “Queue *i*” entry is 0, then the “Queue *i* + 1” entry must be 0;
- if all the queue entries of a node are 0 (meaning that there is no session at the node), then the “Next hop,” “Destination,” and “Channel width” entries must be 0 for this node;
- if the “Next hop” entry of a node is 0 (meaning that there is no session transmitted from this node), then “Channel width” must be;
- because of limited channel width and signal interference, some sessions may be stranded at the current time slot. For example, in Figure 6(a), the “Next hop” and “Channel width” entries at Node 3 are both 0 for the 1.72 Mbits session, and, hence, this session stays at Node 3 at the current time slot and will only be transmitted at later time slots;
- within the same time slot, the total sum of the allocated channel widths for “Channel width” cannot exceed the total spectrum band width, that is, 80 MHz, in this paper.

Take Figure 6(a) as an example for a 6-node network topology for transmission of six sessions: two sessions at Node 1 and one session at each of Nodes 2–5. Figure 6(b) is the encoding for the configuration after finishing the time slot of Figure 6(a), which is explained as follows:

- assume that all the allocated channel widths in Figure 6(a) are large enough to allow transmission of each session;
- the session of size 3.58 Mbits at Node 1 in Figure 6(a) disappears in Figure 6(b) because its destination ID is the same as the ID of the next hop in Figure 6(a); that is, it arrives at its destination after this time slot;
- the session of size 4.71 Mbits in “Queue 2” of Node 1 moves to “Queue 1” and their corresponding destinations are adjusted accordingly;
- there is no action for the session of size 1.72 Mbits at Node 3 because no channel width is allocated to the link adjacent to Node 3;
- three sessions (of sizes 1.13, 6.55, and 0.71 Mbits, resp.) in Figure 6(a) have the same next hop node (Node 3). However, Node 3 has only two empty queue entries (i.e., “Queue 2” and “Queue 3”). In this case, we arbitrarily select two of the three sessions to be transmitted. In this example (see Figure 6(b)), the sessions of sizes 6.55 and 0.71 Mbits arrive at Node 3, while the session of size 1.13 Mbits stays at the original Node 2.

**Figure 7:**
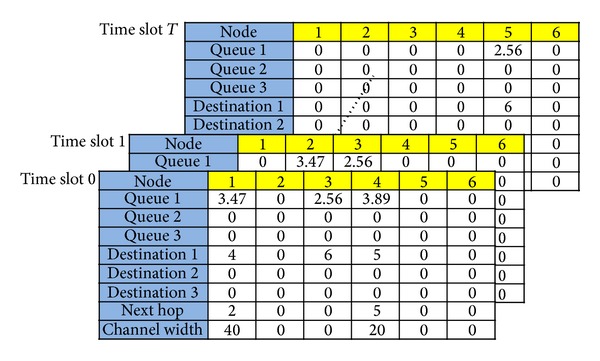
The encoding for a solution of our concerned problem includes the configurations of *T* + 1 time slots.

A complete encoding for a solution of our concerned problem contains configurations of *T* + 1 time slots, as shown in Figure 7. As time slots take turns, the configuration of each time slot determines its own queue and destination entries based on the results from the configuration of the previous time slot and randomly determines its “Next hop” and “Channel width” entries. The same process is repeated until all sessions are transmitted to their final destinations. In choosing the “Next hop” and “Channel width” entries, we need to consider the influences caused by the transmission range to the next hop, the transmission interference constraint, and the variable-width channel allocation. For example, if the transmission distance between nodes is too long, or 40 MHz cannot be used as the maximal channel width, then the 40 MHz option is removed, and one from the remaining options (5, 10, or 20 MHz) is chosen to allocate the channel width. Within each time slot, the allocated channel width and SINR are adopted for the Shannon capacity equation to acquire the system activation time *λ*_*t*_ for each time slot *t*, and finally all the times are summed up to calculate the total system activation time, which is the energy value of the encoded system state.

#### 3.2.2. Generation of the Initial State

In the beginning of the SA algorithm, an initial system state needs to be decided, and this state should match the initial solution and meet all the constraints described in the previous subsection; that is, this system state must permanently maintain the feasibility of the represented solution. Consider to transmit a number of sessions in a network topology, in which the number of nodes, the distance between nodes of the topology, and the size, the source node, and the destination node of each session are known. The steps of generating the initial state are given as follows:

- generate all the queue and destination entries in the configuration of the initial time slot;
- under Constraint (5), one of the nodes to which the session at “Queue 1” can be transmitted is chosen randomly and is assigned to the “Next hop” entry of the session. If the distance *d*_*i*,*j*_ between two nodes is greater than *D*_5 MHz_ (the maximal possible transmission range), then the “Next hop” entry should be chosen again for the session;
- by (1) and Constraint (7), a channel width option is chosen randomly to determine the “Channel width” entry for the session to be transmitted, that is, the one at “Queue 1.” If there are no suitable channel width options, both the “Next hop” and “Channel width” entries for the session are set to 0; that is, the session will be transmitted at later time slots;
- go back to Step (ii) until all the “Channel width” and “Next hop” entries are determined;
- if there are still sessions that have not yet reached their destinations at this time slot, keep their queue and destination entries, and go back to Step (ii) at the next time slot. If all the sessions within this time slot have reached their destination nodes, the transmission process is finished;
- calculate and sum up the system activation time of each time slot to obtain the total system activation time, which is the energy value of the initial state.

#### 3.2.3. Neighborhood Selection

**Figure 8:**
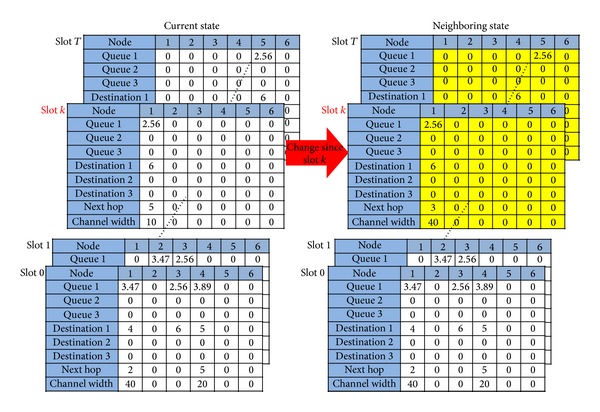
Illustration of neighborhood selection.

This section describes how to search the neighboring state to achieve iterative improvement via the SA algorithm. Consider the current system state that contains configurations of *T* + 1 time slots. We randomly choose a time slot *k*. In the neighboring state as illustrated in Figure 8, the configurations from time slot 0 to time slot (*k* − 1) are the same as those of the current state, while the configurations from time slot *k* to time slot *T* are changed using the the same procedure of generating the initial state in the previous subsection.

Consider the example in Figure 8, in which *k* is chosen as the cut-off point to generate the new neighboring state. In the new neighboring state, the configurations from time slot 0 to time slot (*k* − 1) are kept unchanged as the current state, and the sessions that have not been transmitted to their destination nodes at time slot (*k* − 1) will be transmitted at later time slots. As shown in Figure 8, for the new neighboring configuration, there is a session (of size 2.56 Mbits) at Node 1 at time slot *k* and its destination node is still Node 6. The “Next hop” and “Channel width” entries are then randomly chosen. Form Figure 8, it can be seen that for the new entity, “Next hop” = 3 and “Channel width” = 40 MHz at time slot *k*, unlike the current state, in which “Next hop” = 5 and “Channel width” = 10 MHz at time slot *k*. Following the same procedure, a new neighboring state will be generated after all sessions are transmitted successfully.

Note that the new neighboring state is not necessarily better than the current state and may even have a worse energy value. Thus, an iterative improvement process must be carried out with the SA algorithm to accept an inferior solution and skip the local optimal solution before a superior feasible solution or a nearly optimal solution can be found.

## 4. Experimental Results and Analysis

In order to evaluate the performance of our proposed approach, we implement the proposed algorithm and conduct a variety of experiments. Since our joint RSC problem is new, we first compare our experimental results for a small-scale problem (in which the SINR is not considered) with the optimal solutions generated by the column generation algorithm. Note that column generation is an exact algorithm, which is only suitable for small-scale problems. Then, we evaluate the performance of our SA algorithm for larger-scale problems with the concern on the SINR caused by the transmission range between nodes. Our algorithm is implemented in C++ programming language and runs on an Intel Core i5-3210 M @ 2.50 GHz CPU PC with 8 GB memory to analyze the performance. The parameters settings for our SA algorithm in the experiments are listed as follows: Kotzman parameter *K*_*B*_ = 1; highest temperature *T*_*h*_ = 1000; lowest temperature *T*_*ℓ*_ = 10; cooling constant *γ* = 0.95; and maximum number of iterations *η* = 10000.

### 4.1. Experimental Results for Small-Scale Problems

We continue using the problem instance (where SINR interference is not considered) and parameter settings in [11] as follows: *P*_*o*_ = 1 m Watt, *α* = 2, *N*_0_ = 10^−6^ Watt/MHz, and *β* = 1.3.

**Figure 9:**
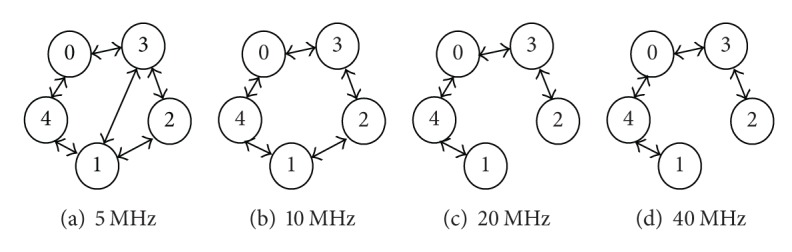
The network topology of adopting the channel width of (a) 5 MHz, (b) 10 MHz, (c) 20 MHz, and (d) 40 MHz.

Recall that the variable-width channel allocation scheme is applied. The network topology considered in [11] using 5 MHz, 10 MHz, 20 MHz, and 40 MHz channel widths is shown in Figures 9(a)–9(d), respectively. As compared with Figure 9(a), the topology in Figure 9(b) does not have link (1, 3), implying that the adoption of 5 MHz channel width can transmit farther. Similarly, for Figures 9(c) and 9(d), the greater the allocated channel width is, the shorter the sessions can be transmitted. Our experiment in this subsection is to transmit the three sessions in Table 1 in this topology.

The problem in the network scenario is solved by our SA algorithm with 5 MHz fixed-width, 40 MHz fixed-width, and variable-width channel allocation schemes, respectively, and the experimental results compared with the optimal solutions generated by the column generation algorithm are given in Tables 2–4. From the result of adopting 5 MHz fixed-width channel allocation scheme in Table 2, our SA algorithm can find a solution with the same solution as the optimal solution generated by the column generation algorithm, though the orders of active links are different. As for the running time, our SA algorithm with the 5 MHz fixed-width channel allocation scheme only takes 3.739 seconds to obtain the solution.

From the result of adopting 40 MHz fixed-width channel allocation scheme in Table 3, the total system activation time found by our SA algorithm is 2.25908 seconds, which is slightly slower than 2.11524 seconds by the column generation algorithm. In this case of using 40 MHz fixed-width channel allocation scheme, the allocated channel width increases and the transmission range decreases, and, hence, more hops are needed so that the SA algorithm is harder to acquire the optimal solution. However, our algorithm can still acquire the nearly optimal solution in 21.949 seconds, showing the computational efficiency of our algorithm. Note that we only compare the minimal and the maximal fixed-width channel allocation schemes with the variable-width channel allocation scheme, because the results of 10 MHz and 20 MHz are between those of 5 MHz and 40 MHz.

From the result of adopting the variable-width channel width scheme in Table 4, the total system activation time found by the SA algorithm is 1.51011 seconds, which is slightly slower than 1.33181 seconds by the column generation algorithm. Compared with the result of adopting the 5 MHz fixed-width channel allocation scheme in Table 2, the total system activation time is 4.66991 seconds faster or shows 76% improvement; compared with the result of adopting the 40 MHz fixed-width channel allocation scheme in Table 3, the total system activation time is 0.74897 seconds faster or shows 33% improvement. Thus, it can be concluded that although our SA algorithm with variable-width channel width allocation scheme may not find the optimal solution as compared to the column generation algorithm, it can still find good-quality feasible solutions, and it is more efficient than the column generation algorithm.

### 4.2. Experimental Results for Larger-Scale Problems

This subsection focuses on the changes of the number of session transmissions in the topologies of different sizes to evaluate the performance difference between variable-width and fixed-width channel allocation schemes. Different from the results in previous subsection, the influences of node locations and transmission ranges between nodes are considered to reflect the impacts of SINR on the channel capacity.

#### 4.2.1. Scenario Test of Transmitting 5 Sessions in a 10-Node Network

**Figure 10:**
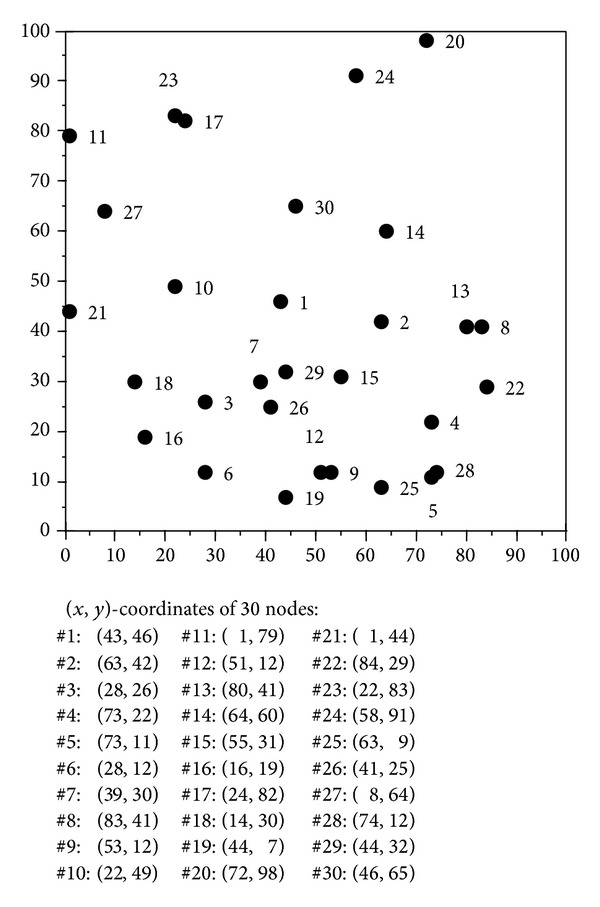
The locations of the 30 nodes in a network.

The network topology considered in this experiment contains 10 nodes on a 100 m × 100 m area, which are nodes #1—#10 in Figure 10. In this topology, we restrict that the *y*-coordinates of all the 10 nodes are no greater than 50, because as the space used for the topology is reduced to half, the probability of* session strain* can greatly decrease. Note that the* session strain* refers to the fact that the initial position of a session happens to be on an outlier node and even though a 5 MHz channel width (the channel width with the ability to transmit sessions to the longest range) is allocated, the session still cannot be transmitted even to the next node, causing the session to strain at the initial node permanently. The problem in this experiment is to transmit the five sessions of different traffic loads in Table 5 in the 10-node network topology.

**Figure 11:**
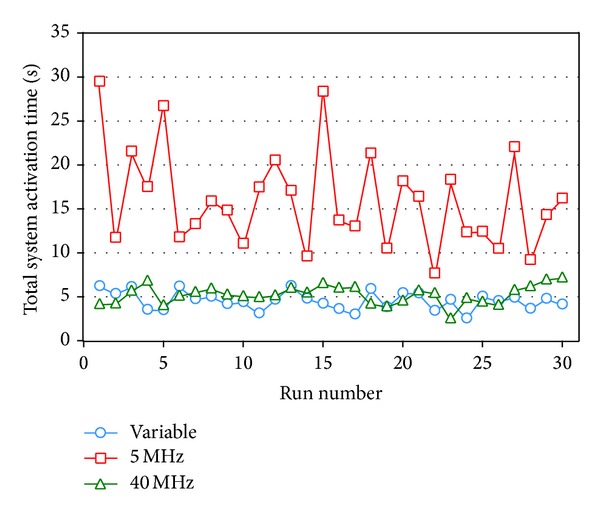
Line chart of the experiment result of transmitting 5 sessions in a 10-node network.

The results of executing 30 runs of our SA algorithm with variable-width, 5 MHz fixed-width, and 40 MHz fixed-width channel allocation schemes are plotted in Figure 11, from which it is obvious to see that the performance of both variable-width and 40 MHz fixed-width channel allocation schemes is better than the 5 MHz fixed-width scheme. The averages of their total system activation times of executing 30 runs of our SA algorithm with the three channel allocation schemes are 4.6255, 16.1476, and 5.3161 seconds, respectively. In average, compared to the 5 MHz fixed-width channel allocation scheme, the variable-width scheme takes about 11.5 seconds less time, or 249% improvement, which is truly remarkable. As for the 40 MHz fixed-width channel allocation scheme, the variable-width scheme takes 0.6906 seconds less time, or 15% improvement.

Although the variable-width channel allocation scheme performs better than the 40 MHz fixed-width channel allocation scheme in average, they still cross with each other in Figure 11 and their performance difference is not immediately discernable. Thus, the *t*-test was conducted to check if the variable-width channel allocation scheme performs statistically better than the 40 MHz fixed-width scheme. The *t*-test result shows that the significance level of Levene's test for equality is 0.964 and greater than 0.05, implying that variance has to be set the same. In the *t*-test with the same means, the significance level is 0.012, which is lower than 0.05. Thus, we can conclude that at the 95% confidence level, the performances of the variable-width and 40 MHz fixed-width channel allocation schemes are significantly different, and the total system activation time of the variable-width channel allocation scheme is clearly shorter than that of the 40 MHz fixed-width scheme.

#### 4.2.2. Scenario Test of Transmitting 7 Sessions in a 10-Node Network

In addition to the five sessions in Table 5, two more sessions in Table 6 are considered in the same network topology in the previous subsection, to evaluate the performance with heavier traffic loads.

**Figure 12:**
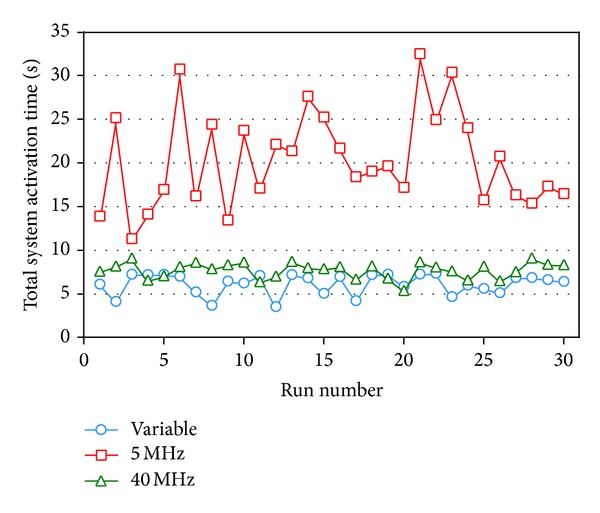
Line chart of the experiment result of transmitting 7 sessions in a 10-node network.

The results of executing 30 runs of our SA algorithm for the same problem of the previous subsection with the addition of transmitting 2 more sessions are plotted in Figure 12, in which the averages of their total system activation times of adopting variable-width, 5 MHz fixed-width, and 40 MHz fixed-width channel allocation schemes are 6.2013, 20.465, and 7.7593 seconds, respectively; that is, all of them are increased as compared with the case of transmitting 5 sessions, and the increasing rates are 34.07%, 26.74%, and 45.96%, respectively. This implies that, in the same topology, the heavier the traffic load is, the more the orthogonal channels are required to transmit more sessions concurrently and reduce the required total system activation time. As shown in Figure 12, the results of the variable-width and 40 MHz fixed-width channel allocation schemes do not significantly cross with each other, and our conducted *t*-test also shows that the significance level is higher, indicating that, as traffic load grows, the performance of the variable-width channel allocation scheme gets better.

### 4.3. Scalability Test of Simulated Annealing Algorithm

The experiment of increasing both the numbers of nodes and sessions is conducted to evaluate performance of our SA algorithm in more complex network topologies with 20 nodes and 30 nodes, respectively (which contains nodes #1—#20 and nodes #1—#30 in Figure 10, resp.). Besides the seven sessions specified in Tables 5 and 6, three additional sessions are added in this experiment to increase the transmission traffic, and their information is shown in Table 7.

**Figure 13:**
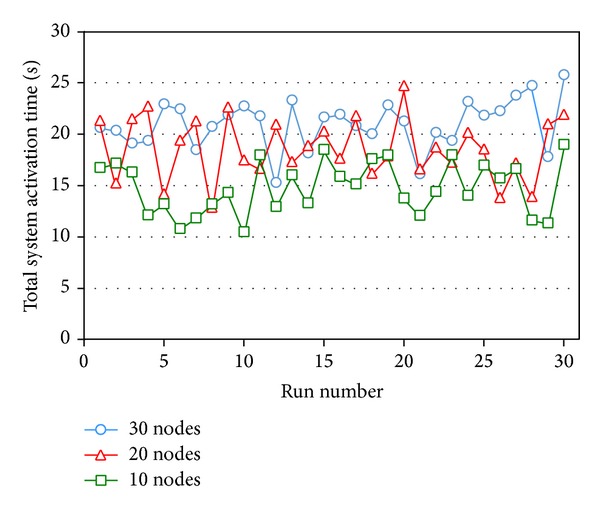
Line chart of the experiment result of transmitting 10 sessions in networks with different numbers of nodes.

The results of executing 30 runs of our SA algorithm to transmit 10 sessions in 10-node, 20-node, and 30-node networks are shown in Figure 13, in which the averages of their total system activation times are 14.8829, 18.6813, and 21.0589 seconds, respectively. In the average, when the number of nodes as well as the level of problem complexity increases, the result is worse, and a better solution cannot be attained as expected with the increase in number of available paths. From Figure 13, the results are different for different number of nodes, indicating that, under the condition that the numbers of sessions to be transmitted are the same, the topology with a higher number of nodes can attain a better result than the topology with a smaller number nodes. Although our SA algorithm may not always obtain the optimal solutions, it can still acquire good-quality solutions within a short time.

## 5. Conclusion and Future Work

This paper has proposed a simulated annealing (SA) algorithm for the joint routing, scheduling, and channel allocation problem in WMNs, in which the variable-width channel allocation scheme is considered to find a delicate balance between mutually conflicting goals, such as concurrent session transmissions and signal interference. A novel encoding design of the SA algorithm is utilized in this paper to illustrate the dynamic transmission process. Our experimental results show that, in comparison to the column generation algorithm for a small-scale problem, in which the SINR is not considered, our SA algorithm can acquire the same or similar solutions as the optimal solutions generated by the column generation algorithm. As compared to fixed-width channel allocation schemes, the variable-width scheme preforms better than the fixed-width scheme. As for the scalability test of our SA algorithm, when the number of nodes grows, the total system activation time also increases. While this result is attributable to the increase in problem complexity and difficulty for identification of the optimal path, causing drop in efficiency, the SA algorithm can still find a good-quality feasible solution. Also, different from the column generation algorithm for exact optimal solutions, our SA algorithm is more efficient to solve the joint problem.

A line of future work is to design the solution encoding mechanism, or integrate our SA algorithm with other artificial intelligent approaches to solve the problem. It would also be of interest to extend the joint problem with mobile nodes or investigate the multiradio multichannel version of the joint problem.

## Figures and Tables

**Table 1:** The size, source, and destination nodes of three sessions.

Session number	Session size	Source	Destination
1	27.4085 Mbits	Node 3	Node 1
2	6.914 Mbits	Node 0	Node 3
3	9.72211 Mbits	Node 1	Node 2

**Table 2:** Result of 5 MHz fixed-width channel allocation.

5 MHz fixed-width	Column generation		Our approach	
Time slot *t*	Active links	Activation time (sec)	Active links	Activation time (sec)
1	(1, 2)	0.467333	(0, 3), (1, 2)	1.61815
2	(3, 1)	4.56187	(3, 1)	4.56187
3	(0, 3), (1, 2)	1.15082	—	—

Total activation time		6.18002		6.18002

**Table 3:** Result of 40 MHz fixed-width channel allocation.

40 MHz fixed-width	Column generation		Our approach	
Time slot *t*	Active links	Activation time (sec)	Active links	Activation time (sec)
1	(0, 3), (1, 4)	0.367965	(0, 3)	0.143846
2	(4, 0)	0.202269	(1, 4)	0.202269
3	(0, 3), (1, 4)	0.202269	(3, 0), (4, 0)	0.570234
4	(3, 0), (4, 1)	0.570234	(0, 4)	0.570234
5	(0, 4), (3, 2)	0.202269	(0, 3), (4, 1)	0.570234
6	(4, 1)	0.570234	(3, 2)	0.202269

Total activation time		2.11524		2.25908

**Table 4:** Result of variable-width channel allocation.

Variable channel width	Column generation		Our approach	
Time slot *t*	Active links	Activation time (sec)	Active links	Activation time (sec)
1	(1, 4), 40 MHz		(1, 2), 10 MHz	0.50337
	(3, 0), 40 MHz	0.158315	(3, 1), 5 MHz	
			(3, 0), 40 MHz	

2	(3, 2), 40 MHz	0.158315	(0, 3), 40 MHz	0.50337
	(4, 1), 40 MHz		(0, 4), 40 MHz	

3	(3, 0), 40 MHz	0.254402	(4, 1), 40 MHz	0.50337
	(4, 1), 40 MHz			

4	(1, 2), 10 MHz	0.030858	—	—
	(4, 0), 40 MHz			

5	(0, 3), 40 MHz	0.159686	—	—
	(1, 2), 10 MHz			

6	(0, 4), 40 MHz	0.570234	—	—
	(1, 2), 10 MHz			

Total activation time		1.33181		1.51011

**Table 5:** Size, source, and destination nodes of five sessions.

Session number	Session size	Source	Destination
1	15.433 Mbits	Node 6	Node 8
2	6.841 Mbits	Node 1	Node 7
3	9.72211 Mbits	Node 3	Node 2
4	4.567 Mbits	Node 2	Node 9
5	17.524 Mbits	Node 8	Node 10

**Table 6:** Besides the five sessions listed in Table 5, two more sessions are added in this experiment.

Session number	Sessions	Source	Destination
6	19.713 Mbits	Node 7	Node 3
7	8.428 Mbits	Node 10	Node 1

**Table 7:** Besides the seven sessions in Tables 5 and 6, three additional sessions are added in this experiment.

Session number	Sessions	Source	Destination
8	4.51 Mbits	Node 4	Node 5
9	16.244 Mbits	Node 5	Node 6
10	8.577 Mbits	Node 9	Node 4
